# Disrupted circadian clocks and altered tissue mechanics in primary human breast tumours

**DOI:** 10.1186/s13058-018-1053-4

**Published:** 2018-10-22

**Authors:** Eleanor Broadberry, James McConnell, Jack Williams, Nan Yang, Egor Zindy, Angela Leek, Rachel Waddington, Leena Joseph, Miles Howe, Qing-Jun Meng, Charles H Streuli

**Affiliations:** Wellcome Centre for Cell-Matrix Research and Manchester Breast Centre, Faculty of Biology, Medicine and Health, University of Manchester, Manchester, M13 9PT UK

**Keywords:** Circadian clocks, Epithelial cells, Breast cancer, Mammographic density

## Abstract

**Background:**

Circadian rhythms maintain tissue homeostasis during the 24-h day-night cycle. Cell-autonomous circadian clocks play fundamental roles in cell division, DNA damage responses and metabolism. Circadian disruptions have been proposed as a contributing factor for cancer initiation and progression, although definitive evidence for altered molecular circadian clocks in cancer is still lacking. In this study, we looked at circadian clocks in breast cancer.

**Methods:**

We isolated primary tumours and normal tissues from the same individuals who had developed breast cancer with no metastases. We assessed circadian clocks within primary cells of the patients by lentiviral expression of circadian reporters, and the levels of clock genes in tissues by qPCR. We histologically examined collagen organisation within the normal and tumour tissue areas, and probed the stiffness of the stroma adjacent to normal and tumour epithelium using atomic force microscopy.

**Results:**

Epithelial ducts were disorganised within the tumour areas. Circadian clocks were altered in cultured tumour cells. Tumour regions were surrounded by stroma with an altered collagen organisation and increased stiffness. Levels of *Bmal1* messenger RNA (mRNA) were significantly altered in the tumours in comparison to normal epithelia.

**Conclusion:**

Circadian rhythms are suppressed in breast tumour epithelia in comparison to the normal epithelia in paired patient samples. This correlates with increased tissue stiffness around the tumour region. We suggest possible involvement of altered circadian clocks in the development and progression of breast cancer.

**Electronic supplementary material:**

The online version of this article (10.1186/s13058-018-1053-4) contains supplementary material, which is available to authorized users.

## Background

Circadian clocks maintain tissue homeostasis during the 24-h day-night cycle. They exist in most cell types and are active after birth. Peripheral clocks are entrained by a central core clock located in the hypothalamus, and are integral to normal tissue function [9, 14, 19, 31, 41]. Cell-autonomous circadian clocks are fundamental in gating cell division, regulating DNA damage responses, and temporally controlling cell metabolism [43]. Altered clocks may contribute to the onset of certain types of tumour, including breast [30, 39]. Interestingly, epidemiological studies indicate that long-term night shift workers have a higher risk of developing breast cancer [22]. Moreover, animal studies reveal an association between clock gene mutations and the initiation, growth rate, and metastasis of mammary tumours [4, 16].

The breast is a regenerative organ that undergoes frequent periods of tissue remodelling [38]. Mouse models have been used to reveal the changes found in mammary gland tissue that are associated with the oestrous cycle, pregnancy, and lactation. During each oestrous cycle, cells proliferate to form alveolar buds on the tertiary side branches and then regress in an ordered fashion [37]. Lobulo-alveolar growth and differentiation takes place in pregnancy, when the epithelial structures expand dramatically to fill the whole fat pad with milk-secreting alveoli [18]. Upon weaning, involution is triggered to remove the milk-secreting cells and return the gland to a non-pregnant state [2]. These developmental processes repeat with each oestrus cycle or pregnancy.

One consequence of the highly proliferative nature of mammary gland biology is that it is subject to abnormalities, which can result in cancer. Breast is the tissue that is most subject to cancer in the human female population worldwide, and causes a high degree of patient mortality [48]. Apart from a small percentage of cancers arising in women with inherited mutations, for example in the *Brca* genes, the cause of breast cancer is poorly understood. One of the biggest risk factors is stromal composition, where women with stroma that has a high mammographic density (MD) have a greater risk of developing cancer [46].

We have shown that circadian clocks are present in the mammary gland, and that they are required for maintaining the tissue stem cell population [53]. Moreover, the breast circadian clock amplitude changes during ageing. Approximately 600 genes are under circadian control in mouse mammary gland, and the oscillation amplitude of the circadian clocks is controlled by the biomechanical stiffness of the tissue stroma.

This is potentially relevant to breast cancer because high MD is linked to stiffer micro-scale stromal tissue [35]. This suggests that a stiffer tissue microenvironment could have an impact in causing cancer. However, it remains unclear whether stromal regions around early human breast tumours are indeed stiffer than those surrounding normal breast tissue, and how a stiffer stroma might promote cancer. One possible mechanism could be through alteration of circadian “time-keeping” clocks that are present in almost all the major body organs, including the breast [4].

There have been a few reports of changes in clock genes/circadian rhythm in immortalized breast tumour cell lines [10, 17, 42, 54]. However, to the best of our knowledge, it has not yet been established if the molecular circadian timing mechanism alters in primary tissue in patients with breast cancer.

The purpose of this study was to investigate whether the breast circadian clock changes during the progression of healthy to cancerous tissue in human patients. Through unique access to tissues from a Manchester patient cohort undergoing mastectomies, we were able to examine tissue structure and circadian rhythms within normal regions of breast tissue and in the early non-metastatic cancers in the same individuals.

We now reveal that circadian clocks, which are present within the normal human breast epithelium, are disrupted in the tumour cells isolated from the same individuals. Moreover, there are alterations in the cellular composition, the organisation and the biomechanical stiffness of tumour stroma, in comparison to the stroma around the normal epithelium of the same patients. Given that the clocks within cells isolated from these individuals are down-regulated in the tumours, we suggest that the altered tumour stroma may contribute to the disrupted clocks.

## Results

### The cellular microenvironment of normal and breast cancer tissue

A cohort of women (aged 46–78 years) was recruited from the Nightingale Centre breast-screening centre (Table 1). These patients were diagnosed with breast cancer, but they did not have metastases. A clinical radiologist assessed the mammograms from each patient and suitable sample areas of normal tissue, at least 4 cm away from the tumour margin, and tumour tissue were identified (Additional file 1: Figure S1). After mastectomy, a clinical histopathologist took biopsies from the highlighted tissue areas in the resected breast for laboratory analysis, and confirmed the absence and presence of neoplasia in normal and tumorous regions.

**Table 1:** Disease status of breast tissue examined in this study

Patient number	Age (years)	Ethnicity	BMI	Tumour details	Histology	Grade	Size (mm)	ER	PR	Her2	Her2 status	Overall tumour phenotype
Patient-1	46	White	33.7	Left breast - lower outer quadrant (LOQ)	IDC	2	23	6	8	1	Neg	ER/PR/Her2-Neg
Patient-2	77	White	36.9	Left breast - upper outer quadrant (UOQ) Mass 29 mm + 2 similar densities USS - 26 mm 3 o’clock - 21 mm	Sarcomastoid/spindle cell/metaplastic carcinoma	3	22					
Patient-3	78		27.8	Right breast - upper half	IDC	3	70	0	0	1	Neg	ER/PR/Her2-Neg
Patient-4	55	white	38.6	Right breast - UOQ. MRI scan	ILC	2	62	8	7	1	Neg	ER/PR/Her2-Neg
Patient-5	69		37.2	Left breast - UOQ 3 cm	Invasive mucinous carcinoma	3	50	5	0	3	Pos	ER/Her2
Patient-6	67	White	34.3	Left Breast - UOQ - 2 o’clock. T2 N0	IDC	2	22	8	7	0	Neg	ER/PR/Her2-Neg
Patient-7	53		34.4	Right breast lobular cancer	ILC, multifocal	2	52	8	7	1	Neg	ER/PR/Her2-Neg
Patient-8	52	White	28.7	Left Breast - UOQ	Lobulated mass IDC	2/3	24	8	0	2 (non-amp)	Neg	ER/Her2-Neg

Details of the age, ethnicity, body mass index (BMI), and tumour status of breast tissue were analysed. We used breast tumour material, plus normal tissue from the same breast that was located at least 4 cm from the tumours

*Abbreviations: ER* oestrogen receptor, *PR* progesterone receptor, *Her2* human epidermal growth factor receptor, *IDC* invasive ductal carcinoma, *Neg* negative, *MRI* magnetic resonance imaging, *non-amp* non amplified

**Fig. 1:**
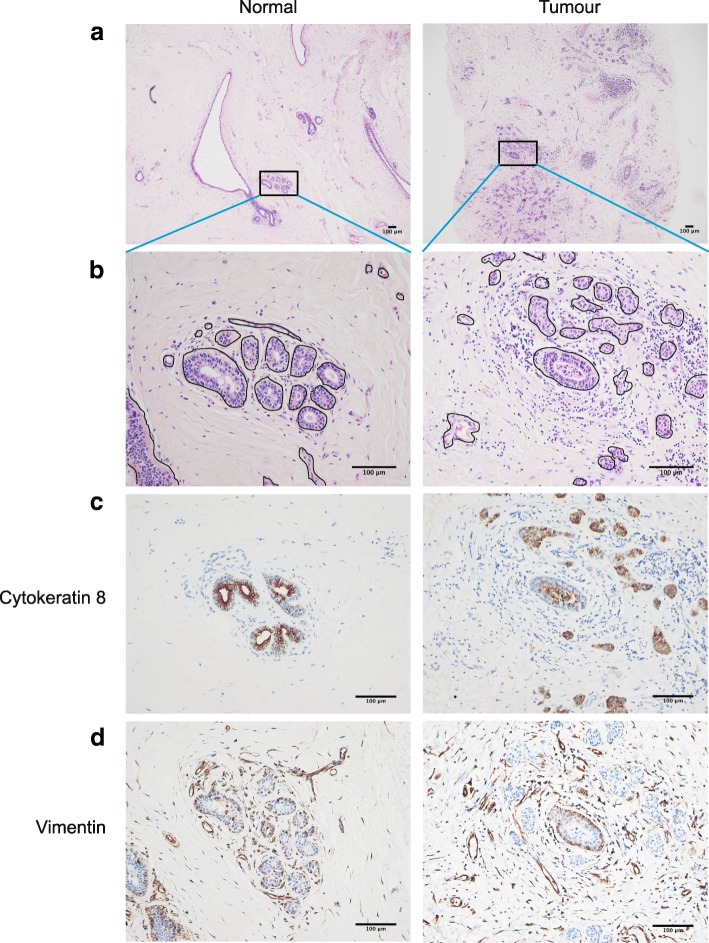
Breast tissue morphology and composition. **a** Representative low power view (× 4) of H&E stained paraffin sections from normal and tumour human breast tissue (patient-6). Additional samples are shown in Additional file 2: Figure S2 and Additional file 3: Figure S3. **b** High magnification (× 20) view of the H&E stained area within the box in the image (**a**). **c** Cytokeratin 8 expression in ductal regions of breast, counterstained with haematoxylin**d** Vimentin expression in similar regions

To identify the ductal and stromal regions, tissue sections from the same individuals were stained with H&E (Fig. 1a; Additional file 2: Figure S2). The epithelia formed ducts with hollow lumens. Ductal structures were also present in the tumour tissue. However, the tumour cells invaded the ducts and filled the lumens, and tumour cells around the ducts formed abnormal growths. Stromal cells surrounded the ducts, and in the tumour tissue they were arranged in arrays of cells (Fig. 1b; Additional file 2: Figure S2).

To determine whether there were any changes within the breast microenvironment of early cancers in comparison to normal epithelia, serial tissue sections corresponding to the H&E-stained regions were immuno-stained for cytokeratin 8 (CK8) (Fig. 1c, Additional file 3: Figure S3). In normal tissue, the luminal layers of ducts were strongly stained for CK8, revealing hollow tubular structures with no cellular obstructions. In malignant tissue, regions of CK8 staining were present in the ducts, confirming the presence of epithelial cells. Transformed cells were also inside ducts and had broken through the ductal walls and were invading the surrounding tissue.

Stromal fibroblasts are actively involved in tumour progression [21]. Serial tissue sections were stained with the fibroblast intermediate filament marker, vimentin (Fig. 1d, Additional file 3: Figure S3). In normal breast, fibroblasts were present in the stroma that neighboured the mammary ducts, while the stroma at a distance from the ducts contained fibroblasts scattered in smaller numbers. Throughout tumour regions, there were more fibroblasts; they were concentrated around ductal epithelia, and their numbers increased in the tumour microenvironment.

These results show that there are differences in structural organisation between normal and malignant tissues from the same breasts, with the tumour epithelium disorganised and infiltrating the stroma. Thus, the organisation and composition of breast tissue change in early malignancy.

### Breast cancer epithelia are altered in cell culture

**Fig. 2:**
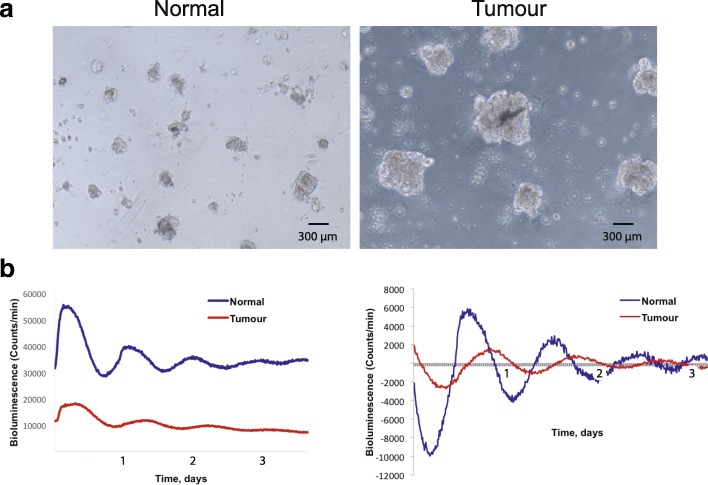
Primary mammary epithelial cells (MECs) in 3D culture**. a** Phase contrast image of MECs isolated from normal and tumour breast tissue from the same patient, after 4 days in 3D culture (patient-4). MECs were plated at the same density but those isolated from tumour tissue formed larger clusters. The data are representative of similar studies from three patients. Scale bar 100 μm. **b** Left, representative *Per2::Luc* traces from cultures of MECs isolated from normal and tumour breast tissue (patient-3). Right, normalisation of *Per2::Luc* activity from normal and tumour MECs. Additional samples are shown in Additional file 4: Figure S4

To determine whether there are any changes in the growth potential of epithelial cells in early cancers in comparison to normal breast, the patient tissues were digested overnight with collagenase, and the epithelial cells were then isolated for cell culture. Primary mammary epithelial cells (MECs), isolated from normal and cancerous regions in the same individuals, were seeded as single cells at the same cell density, and cultured on Matrigel®. The cells isolated from the tumour formed larger clusters than those from normal breast (Fig. 2a). Thus, there are differences in the behaviour of epithelial cells from normal vs tumour regions in the same breasts, when they are cultured on 3D extracellular matrix (ECM).

### Breast cancer epithelia have dampened circadian clocks

We have previously established that the mouse mammary gland and isolated mammary epithelia have autonomous circadian clocks. Robust 24-h rhythms were demonstrated in mammary tissue explants and in primary MECs isolated from mice expressing the luciferase clock (protein fusion) reporter, PER2::Luc [53].

To determine whether there are any changes in circadian rhythms in the epithelia of early breast cancers in comparison to normal tissue, primary human MECs were transduced with a *Per2::Luc* clock reporter via lentiviral infection. The cycling of *Per2::Luc* levels, which faithfully reflects endogenous circadian clock activities, was then examined. The epithelial cells from normal human breast had a robust ~ 24 h *Per2::Luc* rhythm, indicating the presence of strong clock machinery (Fig. 2b). In contrast, MECs isolated from the adjacent cancerous tissue from the same individuals displayed a weakened rhythm, with much lower amplitude, and the rhythm was not sustained. This was the case for all three patients examined, confirming that breast tumours had suppressed circadian clocks (Additional file 4: Figure S4). These results indicate that the circadian clock mechanism is compromised in cancer epithelium, in comparison to that in the normal ductal epithelium of the same individual.

### Collagen is more organised in the periductal stroma of tumour tissue

We have previously shown that the robustness of the breast circadian clock is determined by the stiffness of the extracellular environment [53]. In postmenopausal patients without breast cancer, stromal collagen organisation correlates with ECM stiffness [35]. We therefore hypothesised that the stromal collagen in tumour tissue may be more organised, stiffening the ECM and dampening the circadian clock in the cancerous regions of the same breasts. To investigate the organisation of stromal collagen, sections were stained with Picrosirius Red. This enables the histological abundance of collagen bundles to be detected, and when visualised under polarised light microscopy, the organisation of these collagen fibres can be quantified.

**Fig. 3:**
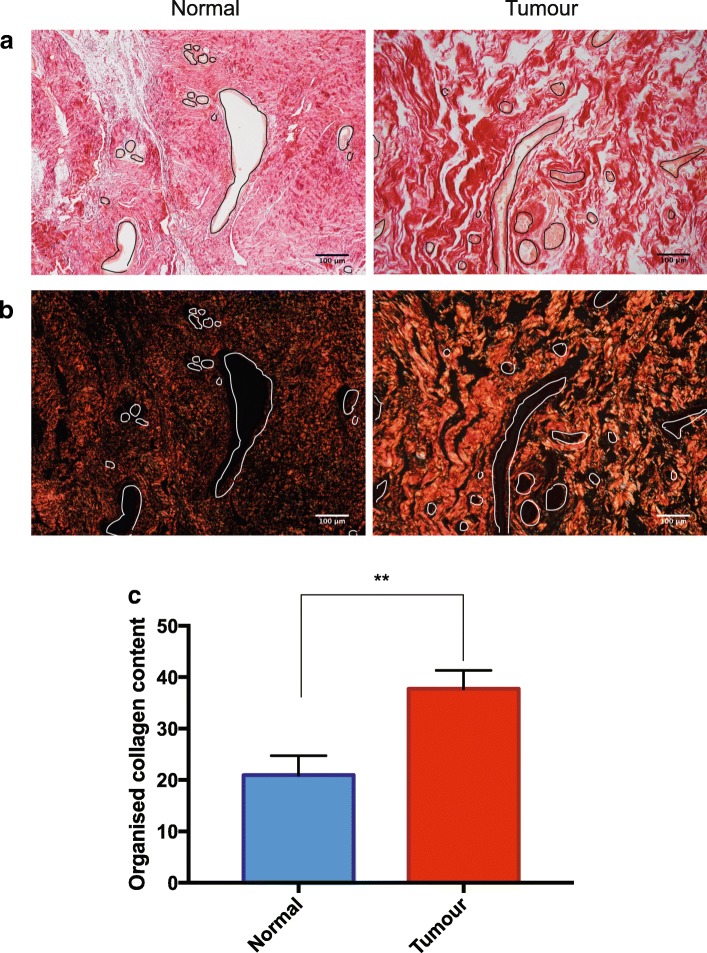
Collagen organisation in periductal stroma. **a**, **b** Picrosirius-Red-stained paraffin sections (patient-6) visualised in bright-field light (**a**) and under polarised light (**b**). Ducts are outlined in black or white. Additional samples are shown in Additional file 5: Figure S5. **c** Percentage organised fibrillar collagen content after quantification, in the normal and tumour tissues: *n* = 6; mean ± SEM; unpaired *t* test

Both normal and tumour tissue contained significant levels of collagen (Fig. 3a). However, when viewed through perpendicular polarizing microscopy, most of the tumour areas showed considerably more organized collagen bundles (Fig. 3b; Additional file 5: Figure S5). A semi-quantitative estimate of the organised collagen content, determined by the percentage of collagen visible under polarised light, in relation to the total collagen present, showed more fibrillar collagen-I in the tumour regions (Fig. 3c). Thus, in most cases the collagen adjacent to epithelial ducts is more highly organised in cancerous regions than in those adjacent to normal tissue from the same patient.

### Tumour stroma is stiffer than that of normal breast

We utilised atomic force microscopy (AFM) to determine whether the altered collagen organisation seen in tumours led to stiffened ECM within the stromal regions of the tissue. Indentations were made on 5 μm cryosections, in regions of stroma adjacent to mammary ducts in both the normal and tumour tissue from the same patients (Additional file 6: Figure S6). In each tissue, 2500 indentations were made, enabling analysis of data point distributions.

**Fig. 4:**
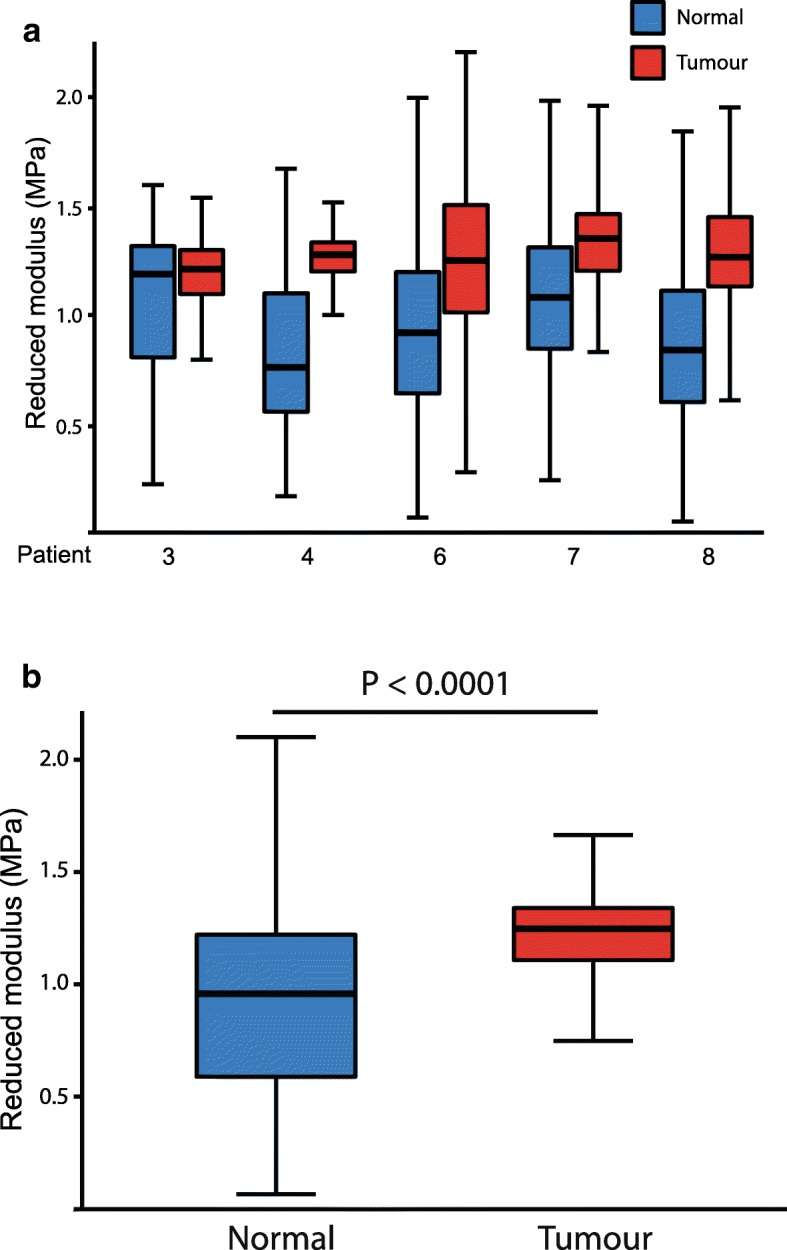
Micromechanical stiffness of periductal stroma. **a** Mean reduced modulus for periductal regions of the stroma in normal (blue) versus tumour (red) tissue sections from five patients. The regions analysed are shown in Additional file 6: Figure S6. **b** Mean reduced modulus for periductal regions of the stroma in tumour tissue compared with normal breast tissue (in all five patients assessed). Tumour periductal stroma (reduced modulus = 1.21 MPa) was significantly stiffer than normal periductal stroma (reduced modulus = 0.90 MPa, *p* < 0.0001, *n* = 5)

There was a positive (right) shift in the distribution of reduced moduli (a measure of tissue stiffness) in tumour tissue compared to the normal breast (Fig. 4a). Our data revealed that there was an average 30% increase in stromal stiffness adjacent to the tumour in comparison to the normal areas (Fig. 4b). Thus, breast tumours have a stiffer ECM adjacent to the epithelial regions than the normal tissue in the same patients.

### The profile of clock genes is deregulated in breast cancer

Previous studies have identified PER1 and PER2, components of the negative arm of the molecular clock, as tumour suppressor genes [24]. A significant decrease in PER1 and PER2 expression has been demonstrated in sporadic and familial primary breast tumours when compared to normal breast tissue [11]. However, few studies have examined the expression of positive clock factors (BMAL1 and CLOCK) in human breast cancer, largely due to the lack of reliable antibodies.

To gain an insight into how breast circadian clocks are altered in cancerous tissue in comparison to the normal breast in the same patients, quantitative PCR (qPCR) of endogenous clock genes (*Bmal1*, *Clock* and *Nr1d1*) was performed in the resected specimens.

**Fig. 5:**
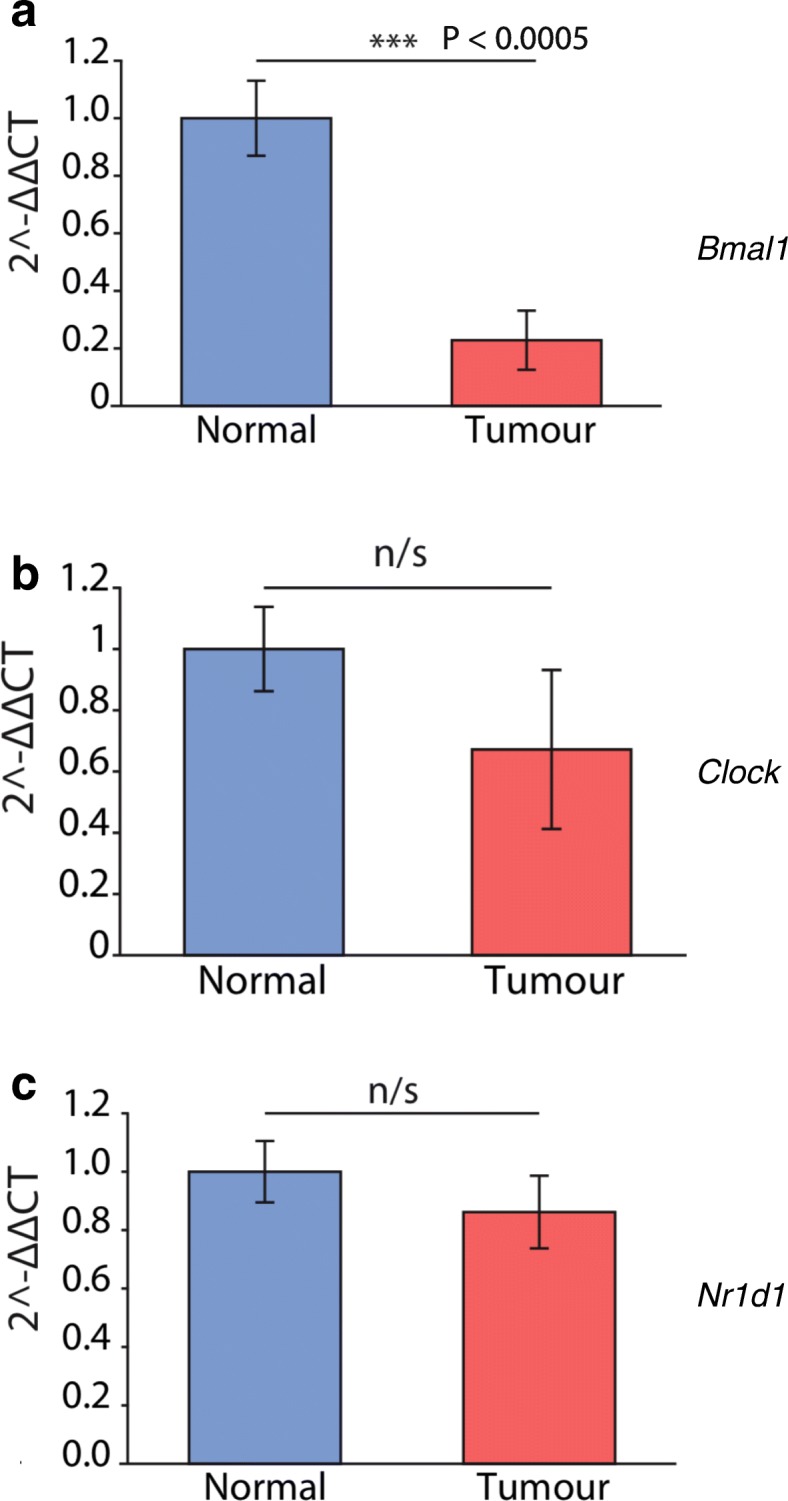
Clock genes expressed in normal and tumour breast tissue. Representative quantitative PCR levels in breast tumour material, plus normal tissue from the same breast that was located at least 4 cm from the tumours. Here, samples from eight separate patients with invasive lobular and invasive ductal carcinoma were used. In each case, RNA levels were normalised to a β-actin loading control, with ∆∆ cycle threshold (CT) quantification of expression. **a** *Bmal1*; **b** *Clock*; **c** *Nr1d1*

Both the normal and cancerous regions expressed *Bmal1* messenger RNA (mRNA). In each case, the levels of *Bmal1* mRNA expression was stronger in normal tissue in comparison to tumour tissue from the same patient (*p* < 0.0005, *n* = 8) (Fig. 5a). In contrast to *Bmal1*, the expression of *Clock* (the binding partner of *Bmal1*) and *Nr1d1* (the negative transcriptional regulator of *Bmal1*) was more variable and showed no consistent changes (Fig. 5b, c).

Our findings of consistent reduction of *Bmal1* mRNA levels, together with reports from others of altered PER proteins, confirms that the intrinsic feedback machinery regulating clock expression is disrupted in early breast cancer [11]. Thus, the altered clock dynamics that we observed in primary cultures of normal versus cancerous tissue reflects different levels of circadian regulators in cancerous breast tissue in patients.

## Discussion

### Overall conclusions

Cell-autonomous circadian clocks are fundamental in cell fate and functions [43]. Disrupted circadian rhythms have been suggested as a risk factor for the development of breast cancer [39]. However definitive evidence linking clock dysregulation with tumour progression in humans is missing [23, 50]. This is partly because there has been a lack of direct comparison of circadian clock properties between cells from tumorous and non-tumourous regions of the same patients. Our study has now compared primary tumours to regions of normal breast within the same individuals.

By monitoring rhythmic activities of a clock gene reporter (*Per2*::luciferase), we discovered that circadian clocks in cancerous epithelium have compromised clock machinery. To the best of our knowledge, this is the first demonstration of mammary clock disruption in epithelial cells freshly isolated from primary tumours of patients with breast cancer. Our data support the hypothesis that the circadian rhythm is suppressed in breast cancer.

### Compositional and mechanical changes in the stroma of breast cancer

By staining breast tissue sections with H&E, cytokeratin and vimentin, we identified distinct structural and compositional changes to the breast tumour microenvironment. In cancerous breast tissue, malignant epithelial cells invade the duct and infiltrate the stroma. In addition, there is an accumulation of fibroblasts within the tumour region. Fibroblasts are key stromal cells that contribute to the interaction of MECs with their associated stroma [6]. This occurs through secretion of ECM components such as collagen and growth factors, enabling the stroma to actively participate in malignant epithelial cell transformation [28]. Our future work will examine how aberrant stromal-tumour cell interactions contribute to the maintenance of a tumour microenvironment.

We also found mechanical changes in the microenvironment of cancerous breast tissue, and suggest that altered stromal stiffness contributes to tumour formation. We know that mammographic density represents the second highest risk factor for breast cancer, and have shown that stiffness is related to differences in collagen organisation [35]. Very little is known about the molecular basis for how different collagen-I networks are formed in either normal or cancer-associated stroma. However, our data suggest that tumour stroma has increased fibrillar collagen organisation compared to matched non-tumour tissue. Indeed, others have shown that the stromal collagen network is reorganised in early desmoplastic tumour progression [3, 29]. Although alterations in stromal organisation and stiffness during tumour progression have been well-characterised, the pathways orchestrating these changes are less understood [1]. Breast tumour-associated fibroblasts over-express lysyl oxidase, stiffening the matrix and leading to increased tumour cell invasion [20]. Lysyl oxidase cross-links collagen fibres, and changing its levels alters collagen organisation, matrix stiffness and the metastatic potential of cell lines [12]. This may be coordinated by hypoxia inducible factors, indeed silencing these factors alters fibrillar collagen synthesis, deposition and degradation in MDA-MB-231 cells [44, 45]. We are currently elucidating the effect of these pathways on collagen deposition in the peri-tumour stroma by examining protein expression with mass spectrometry.

### Altered circadian clocks in breast cancers

Breast epithelium contains circadian clocks, but their amplitude declines during ageing when the stromal areas surrounding the epithelium become stiffer [4, 53]. We now show that the stroma adjacent to human breast tumours is significantly stiffer than it is close to normal epithelium in the same individuals. Given that extracellular stiffness dampens the epithelial circadian clock, our findings begin to explain how the clock may become reduced in breast cancer. However, there are varying explanations of which clock components become altered in cancer to suppress the clock itself.

### A possible role of altered BMAL1 in breast cancer

Our data at the RNA level reveals that in breast cancers some genes contributing to core clock mechanisms, such as that encoding BMAL1, become reduced. BMAL1 is a key circadian transcription factor with central roles in fine-tuning the behaviour of epithelial cells. Currently, we do not know if altered levels of clock gene expression have direct functions in breast cancer or if they are simply correlative. Our future studies will use overexpression and gene deletion approaches in both culture and mouse models to determine their downstream transcriptional targets in breast epithelia, and their role in 3D models of cell growth and survival.

We think it likely that altered stoichiometry of clock factors contributes to altered clocks both in primary tumour cell culture and in the breast itself [8, 32]. Given that circadian dimers such as BMAL1-CLOCK are involved with chromatin opening and transcription of downstream genes, this would not be surprising [36]. Indeed, loss of coordinated *Per3*/*Cry2* gene expression is associated with worse prognosis, particularly in patients with less oestrogen receptor (ER) and with human epidermal growth factor receptor 2 (Her2) amplification [7]. In future we aim to determine whether alterations in the levels of specific clock RNAs, and their encoded proteins, are linked to different types of breast cancers, such as the ER, progesterone receptor (PR)-positive or triple-negative cases. Moreover, further studies on the control of clock gene expression in breast cancer by methylation, is also warranted [33].

Previous work on pancreatic cancer demonstrated that BMAL1 expression was lower in malignant tissues than in tissue from non-cancerous controls [26], as we have also found in malignant breast tissue. BMAL1 was proposed to have an inhibitory effect on cell cycle progression and invasion, suggesting it is protective in this type of cancer. In contrast, over-expression of BMAL1 contributes to the aggressiveness of malignant pleural mesothelioma, with knockdown of BMAL1 suppressing cell proliferation [15]. Consistent with this study, agonists of REV-ERB, which is the main suppressor of *Bmal1* transcription, are lethal to cancer cells and oncogene-induced senescent cells [49].

Altered BMAL1 also influences the development of squamous tumours [25]. In the U2OS osteosarcoma line, BMAL1 over-expression induces apoptosis, while in CA46 lymphoma cells it increases apoptosis and reduces proliferation, leading to smaller tumours when the cells are injected into mice [5]. Stromal cells are the origin of osteosarcoma cells, and we have examined clocks in stromal cells [52]. We found that stromal clocks are regulated oppositely to those in epithelial cells in normal tissues, so it will be interesting to determine possible alterations of clock gene expression in the normal and tumour-associated stromal regions of breast tissue.

## Conclusion

We examined tumour and adjacent normal regions of breast tissue in patients with cancer, and discovered that circadian clocks are suppressed in the tumour epithelium. We also found that there are alterations in the cellular composition, the organisation and the biomechanical stiffness of tumour stroma. The implication is that there is a novel link between ECM stiffening and the control of circadian rhythms in early human breast cancers. This is the first step towards understanding how microenvironmental remodelling alters the circadian clock in breast cancer. At this stage, we still do not know whether the reduction of circadian rhythm is just a consequence of tumorigenesis, or an early event in the establishment of breast cancer. Further investigation into the relationship between cell-matrix interactions and circadian clock gene expression will provide a new understanding about how breast cancer initiates and progresses.

## Methods

### Reagents

We used foetal bovine serum (Labtech), DMEM (Lonza), Matrigel (Corning), insulin, hydrocortisone, epidermal growth factor (EGF), cholera toxin, trypsin-EDTA, bovine pituitary extract (BPE), antibiotic antimycotic solution, collagenase D and hyaluronidase (Sigma).

### Cohort and breast tissue samples used in this study

A radiologist assessed digital mammograms, and putative areas of tumour and normal tissue were identified and then verified by a histopathologist. These areas were excised following mastectomy and transported on ice to the laboratory. Tissue samples were bisected, one part being used for primary culture of epithelial cells and the other for histological analysis, stromal composition and assessment of stiffness and the levels of circadian clocks.

### Human mammary tissue dissection and epithelial isolation

Biopsies were kept in chilled RPMI medium until ready for digestion, then MECs were isolated [47]. The tissue was chopped manually with a scalpel for 10 min and placed into a conical flask containing collagenase mix (3 mg/ml collagenase, 0.7 mg/ml hyaluronidase and 0.2 mg antibiotics), then digested overnight at 37 °C with constant agitation. The following morning, the digest mix was transferred into a Falcon tube and left for 5 min to allow separation, the layer of fat was removed and the sample centrifuged at 1500 rpm for 5′. The resulting pellet was kept on ice whilst the supernatant was transferred into a new Falcon tube and centrifuged for a further 5′, washed twice and resuspended in 10 ml DMEM and 10% FBS and was left for up to 3 h on a 10 cm dish to deplete fibroblasts. The medium containing unbound cells was aspirated, centrifuged at 1000 rpm for 5′, the pellet was resuspended in human growth medium and cultured in complete medium (mammary epithelial cell growth medium (MEBM) supplemented with 52 μg/ml BPE, 5 μg/ml insulin, 500 ng/ml hydrocortisone, 10 ng/ml human EGF and 10% fetal calf serum (Biowittaker). All cells were cultured at 37 °C with 5% CO2.

### Tissue architecture

A portion of tissue sample was fixed in 4% paraformaldehyde in PBS before processing overnight using an automated tissue processor (Shandon Citadel 2000). Samples were embedded in molten paraffin wax using a Leica EG1150H embedding station, sectioned at 3 μm, and mounted onto positively charged slides using a Leica RM 2155 microtome. Sections were dewaxed, rehydrated through a descending alcohol gradient into distilled water, then stained with H&E using a Thermo Fisher Scientific Shandon Varistain 24–4 autostainer, then mounted in DPX mounting medium (Sigma, MO, USA) and left to dry overnight before imaging using an Olympus Slide Scanner.

### Immunohistochemical analysis

Tissue sections were dewaxed and rehydrated through a descending alcohol gradient, then immersed in boiled 10 mM sodium citrate, 0.005% Tween 20, pH 6.0 for 20 min to expose antigen sites. Endogenous peroxidase activity was quenched with H2O2 before staining with primary antibody (diluted in PBS with 0.1% Triton-X 100 and 3% goat serum) overnight at 4 °C in a humidified chamber, then biotinylated secondary antibody was applied (Vector Laboratories, CA, USA) for 2 h at room temperature (RT), and streptavidin-conjugated-Horseradish Peroxidase (Sigma, MO, USA) was added for 1 h. Chromogenic detection was with 3,3-diaminobenzidine (DAB) in the presence of H2O2 for 5 min, slides were counterstained for 2 min in Methyl Green (Sigma, MO, USA), washed in H_2_O or water dehydrated and mounted in DPX mounting medium. The images were viewed using an Olympus Slide Scanner.

### RNA expression

Breast tissue from normal and tumour areas was selected from a subset of patients in the study previously described. Tissue pieces were homogenized using a TissueRuptor II (QIAGEN), and mRNA extracted using an RNeasy Micro Kit (QIAGEN) according to the manufacturer’s protocol. For qPCR, RNA concentrations were determined using a NanoDrop Lite (Thermo Fisher), and equal amounts of RNA were converted to complementary DNA (cDNA) using the High-Capacity cDNA Reverse Transcription Kit (Thermo Fisher). TaqMan-based qPCR was performed using a StepOnePlus Real-Time PCR System (Thermo Fisher) with FAST Blue qPCR MasterMix (Eurogentec). TaqMan primers and probes were purchased from Applied Biosystems (Thermo Fisher). The following probe IDs were used: Actb Hs99999903_m1, Bmal1/Arntl Hs00154147_m1, Clock Hs00231857_m1, Nr1d1 Hs00253876_m1.

### Real-time recording of clock activities

Cells were synchronised an hour before recording with 100 nM dexamethasone (Sigma, MO, USA). Cells were then washed and cultured in warmed HEPES-buffered and sodium bicarbonate-buffered recording medium containing luciferin and sealed with UV-irradiated vacuum grease and 40 mm glass coverslips (Thermo Fisher Scientific, MA, USA). Dishes were placed into photomultiplier tubes (PMTs) or into LumiCyclr apparatus (Actimetrics, IL, USA). Bioluminescence was measured with LumiCycle software and curves were normalised using a 24 h moving average baseline correction.

### Lentiviral packaging

HEK293FT cells were co-transfected with the transfer vector pLV-*Per2-luc* and the packaging plasmids pMD2-VSV-G, pMDLg/pRRE and pRSV-REV [34] using the calcium phosphate method on day 1 [27]. Medium was changed on day 2, collected and changed on day 3, collected on day 4 and then the supernatants were combined and centrifuged at 200 g for 5 min. The resulting supernatant was then centrifuged at 2000 g for 40 min using Vivaspin 20 Centrifugation Columns (Sartorius AG, Göttingen, Germany). The fraction remaining in the column was stored at − 80 °C or used immediately.

### Transducing primary human MECs with a *Per2::Luc* clock reporter

For lentiviral transduction, human MECs were grown to 80% confluence in 35 mm dishes. Cells were transduced with 50 μl lentiviral particles, and the medium was changed to fresh medium after 16 h. For cells transduced with the *mPer2::luc* reporter, this was changed to recording medium and recorded for bioluminescent activity in PMTs.

### Real-time recording of clock activities

Cells cultured in 35 mm dishes were synchronised with 100 nM dexamethasone. Prior to placing in the Lumicycle, normal culture medium was removed and rapidly replaced with pre-warmed HEPES-buffered and sodium bicarbonate-buffered recording medium. The bioluminescence values for all cell types were recorded in this medium. Culture dishes were sealed with coverslips and vacuum grease and placed into the LumiCycle or photomultiplier devices. Baseline subtraction was performed using a 24 h moving average algorithm.

### Collagen organisation

Tissue sections were rehydrated through a descending alcohol gradient into distilled water before staining with Picrosirius Red (Sigma, MO, USA) for 1 h. Sections were washed twice in 5% HAc solution and gently blotted with filter paper to remove any excess stain. Slides were dehydrated, cleared through an ascending alcohol gradient into xylene, before mounting in DPX mounting medium, dried overnight and then imaged on an Olympus Slide Scanner under normal and polarised light. Collagen organisation was assessed from the Picrosirius red staining [40, 51]).

### Atomic force microscopy

Breast tissue was embedded in optimal cutting temperature cryo-sectioning medium in a 2.5 cm mould at − 20 °C, sectioned at 5 μm using an HM560 automated cryostat and stored at − 80 °C. Cryosections were air-dried overnight at RT then examined by AFM. Micro-indentation was carried out using 5 μm cryo-sections and a Nanowizard 4 AFM (JPK, Cambridge, UK) mounted onto an Axiovert T1 inverted optical microscope (Zeiss, Cambridge, UK) fitted with a spherically tipped cantilever (nominal radius and spring constant of 1 μm and 3 Nm^− 1^, respectively: Windsor Scientific Ltd., Slough, UK,) running SPM software v 8.15 (JPK, Cambridge, UK). The local reduced modulus was determined for each of 2500 points in a 50 × 50 μm region, indented at a frequency of 1 Hz. The extend curve was used in conjunction with a contact-point-based model to calculate the reduced modulus for each indentation [13]. Post hoc analyses of force curves were performed using SPM analysis software v 1.40 (JPK, Cambridge, UK), whereby a baseline correction was applied to each curve before a force fit was applied using the Herzian (spherical) model and a maximum force fit of 70%. Once all force curves had been generated, quality control was applied, whereby any force values falling more than two standard deviations away from the mean value were discarded to account for failed indents (fewer than 10% of force curves).

### Statistical analysis

Statistical analysis was done using Microsoft Excel or GraphPad PRISM Data Analysis software. Statistical significance was determined by Student’s *t* test for paired samples when comparing two groups. One-way analysis of variance (ANOVA) was used when comparing more than two groups. Differences between samples were significantly different when *p* = < 0.05. For all graphs shown, error bars represent +/− standard error of the mean. The means have 1–4 asterisks centred over the error bar to indicate the relative level of the *p* value: *p < 0.05, **p < 0.01, ***p < 0.001 and ****p < 0.0001.

## Additional files

Additional file 1: **Figure S1.** Breast tissues used in this study. Mammograms of patients examined in this study. The regions used for analysis were visually alike in most of the patients, and are outlined - red is tumour tissue, while green is normal. (PDF 698 kb)

Additional file 2: **Figure S2.** Histology of tissues used in this study. Histology of normal and tumour regions obtained for this study. In each case the normal regions were 4 cm or more away from the primary tumours in the same breasts. (PDF 41755 kb)

Additional file 3: **Figure S3.** Cytokeratin and vimentin staining of the tissues used in this study. Cytokeratin 8 and vimentin staining of normal and tumour regions used in this study. (PDF 144000 kb)

Additional file 4: **Figure S4.** Circadian clocks in breast tissue**.** Left - representative *Per2::Luc* traces from cultures of MECs isolated from the normal and tumour regions of patients with breast cancer. Right - normalisation of *Per2::Luc* activity from normal and tumour MECs. (PDF 287 kb)

Additional file 5: **Figure S5.** Collagen organisation in normal and tumour stroma. Picrosirius-Red-stained paraffin sections visualised in bright-field or polarised light. Samples of normal (left) and tumour (right) tissue from the same individuals are shown in each case. Ducts are outlined in black and white. (PDF 82243 kb)

Additional file 6: **Figure S6.** Stromal regions analysed by AFM. H&E staining of the normal and tumour regions of breasts from each individual that were examined by AFM (see Fig. 4a). The black squares represent the exact regions that were analysed. (PDF 36538 kb)

## Abbreviations

AFM: Atomic force microscopy
BPE: Bovine pituitary extract
DAB: 3,3-Diaminobenzidine
DMEM: Dulbecco’s modified Eagle’s medium
ECM: Extracellular matrix
EGF: Epidermal growth factor
ER: Oestrogen receptor
FBS: Foetal bovine serum
H&E: Haematoxylin and eosin
Her2: Human epidermal growth factor receptor 2
MD: Mammographic density
MEC: Mammary epithelial cell
mRNA: Messenger RNA
PBS: Phosphate-buffered saline
PMT: Photomultiplier tube
PR: Progesterone receptor
qPCR: Quantitative PCR
