# Supplementary figures and images for: Disrupted circadian clocks and altered tissue mechanics in primary human breast tumours
Source: Breast Cancer Res. 2018 Oct 22;20:125. doi: 10.1186/s13058-018-1053-4 (PMC6198506; doi:10.1186/s13058-018-1053-4)

Patient-1

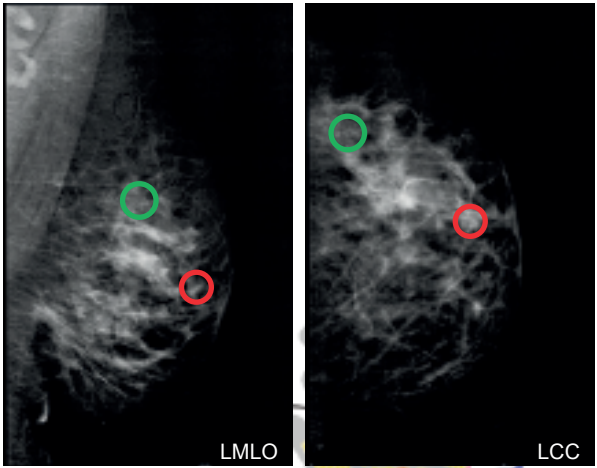

Patient-5

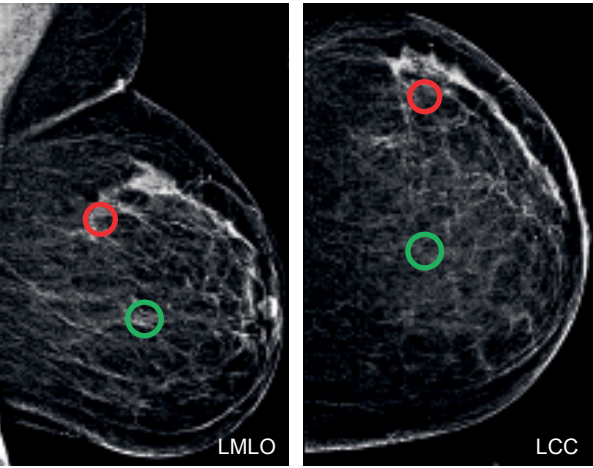

Patient-2

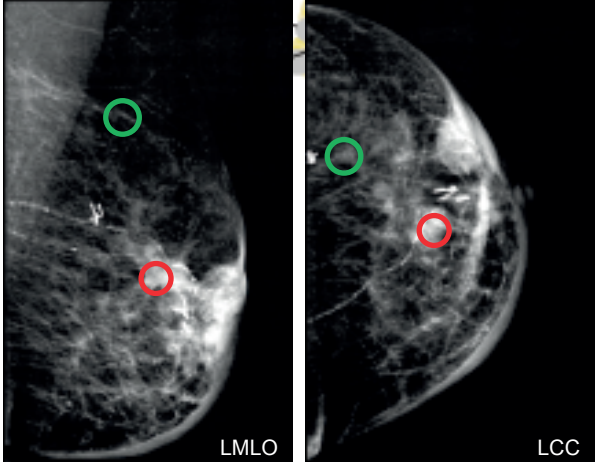

Patient-6

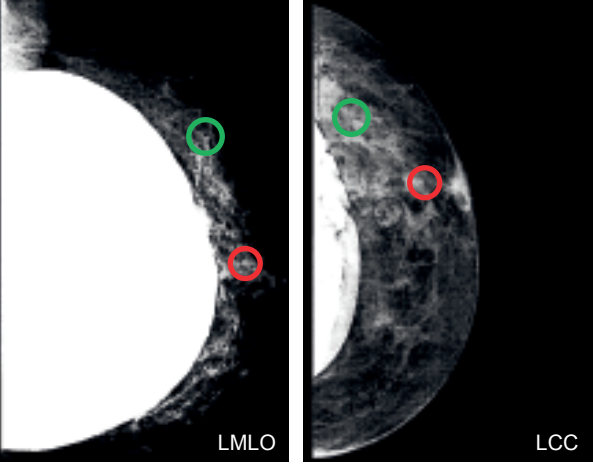

Patient-3

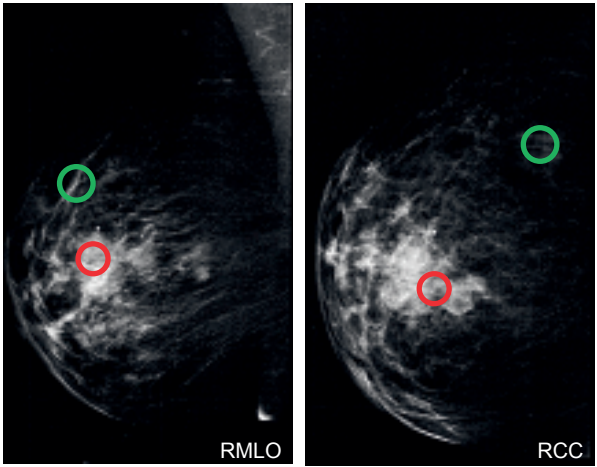

Patient-7

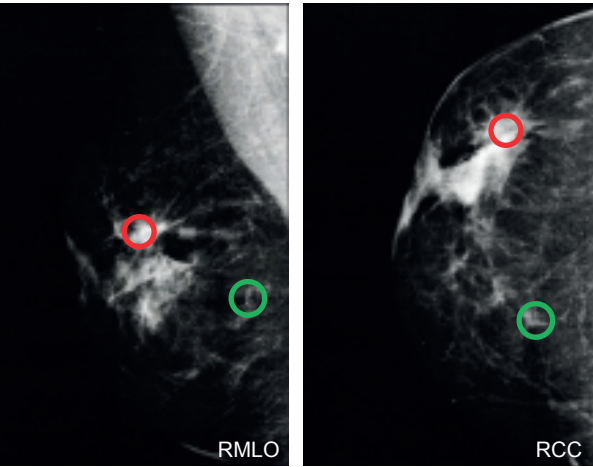

Patient-4

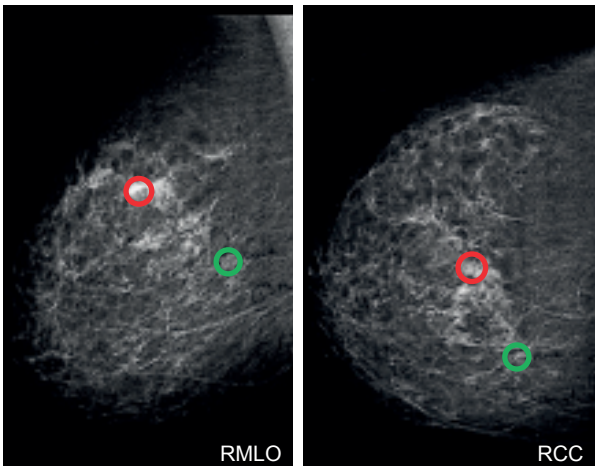

Patient-8

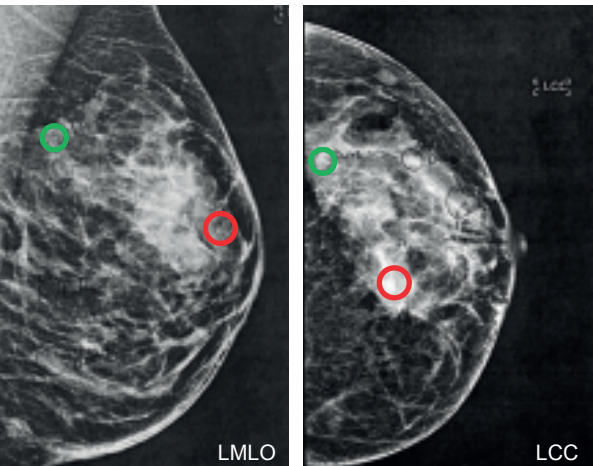

○ Normal  
○ Tumour

Supplement: Supplementary file 1 — Figure S1. Breast tissues used in this study. Mammograms of patients examined in this study. The regions used for analysis were visually alike in most of the patients, and are outlined - red is tumour tissue, while green is normal. (PDF 698 kb) [file 13058_2018_1053_MOESM1_ESM.pdf]

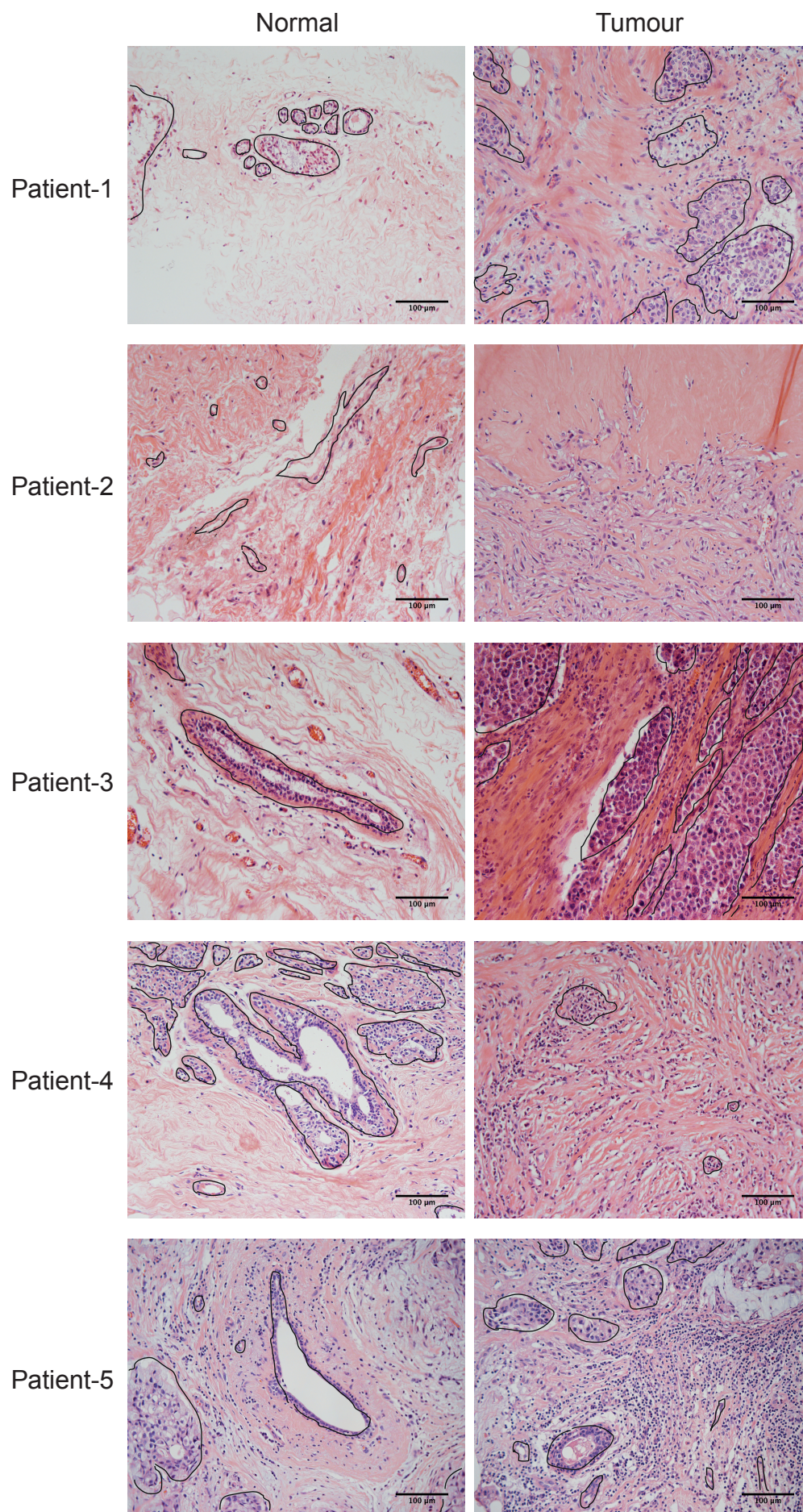

Broadberry Supplementary Figure 2

Supplement: Supplementary file 2 — Figure S2. Histology of tissues used in this study. Histology of normal and tumour regions obtained for this study. In each case the normal regions were 4 cm or more away from the primary tumours in the same breasts. (PDF 41755 kb) [file 13058_2018_1053_MOESM2_ESM.pdf]

CK8

Vimentin

Patient-1

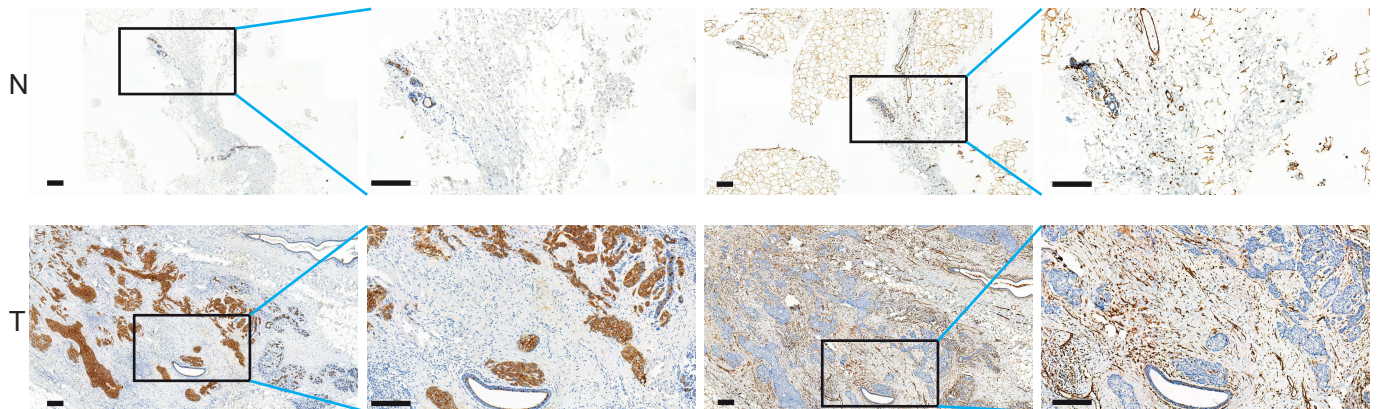

Patient-2

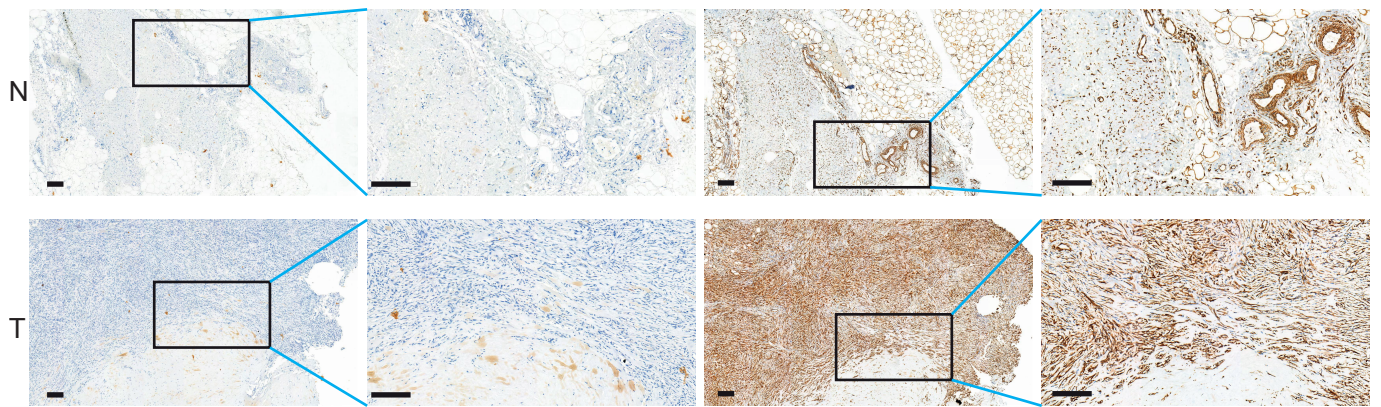

Patient-3

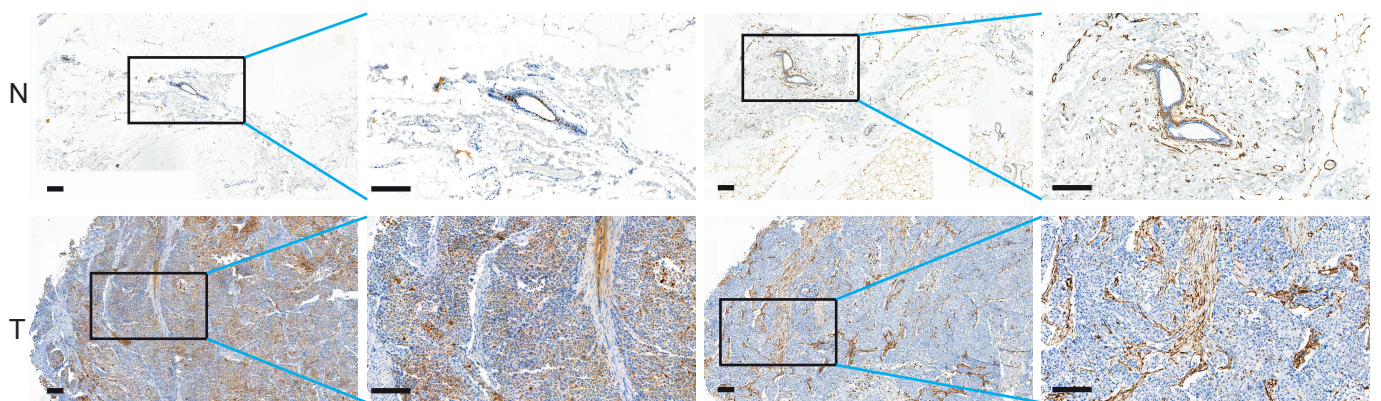

CK8

Vimentin

Patient-4

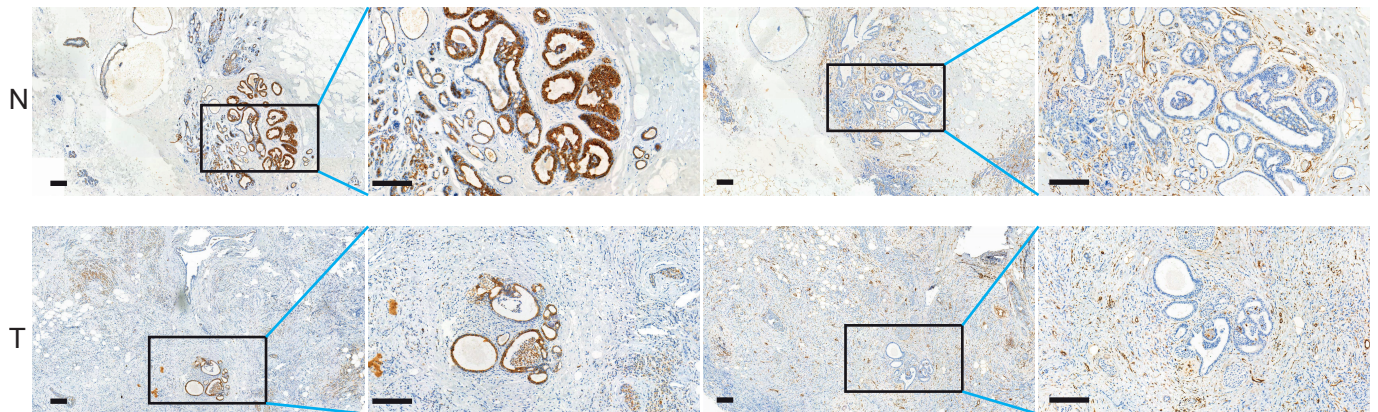

Patient-5

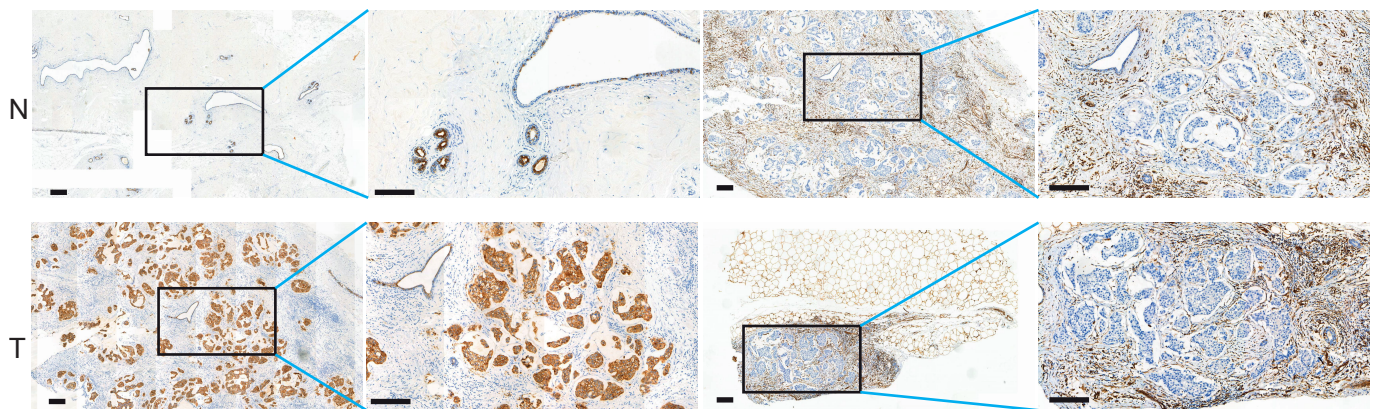

Supplement: Supplementary file 3 — Figure S3. Cytokeratin and vimentin staining of the tissues used in this study. Cytokeratin 8 and vimentin staining of normal and tumour regions used in this study. (PDF 144000 kb) [file 13058_2018_1053_MOESM3_ESM.pdf]

Patient-1

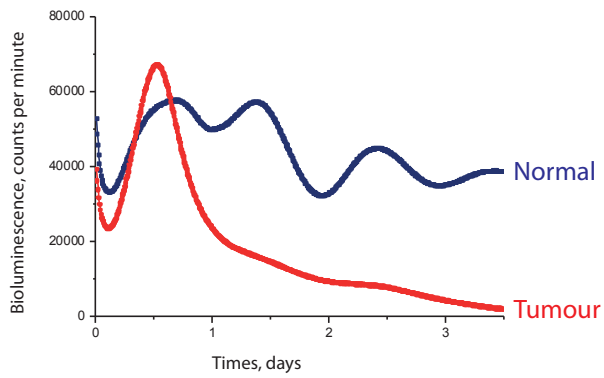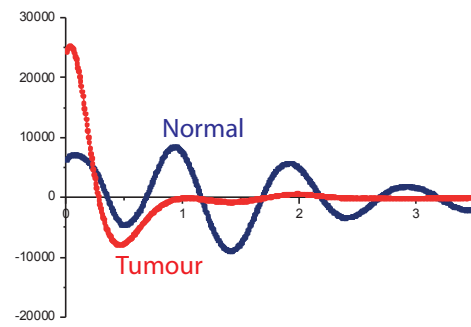

Patient-4

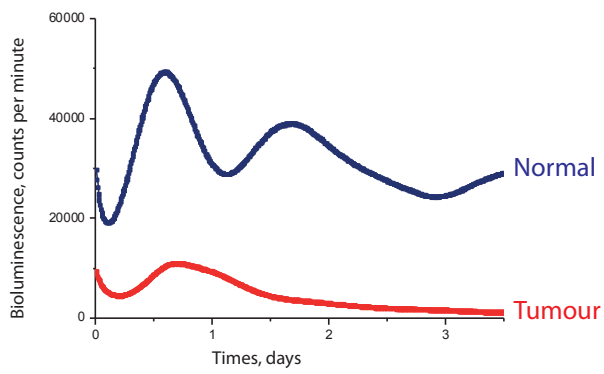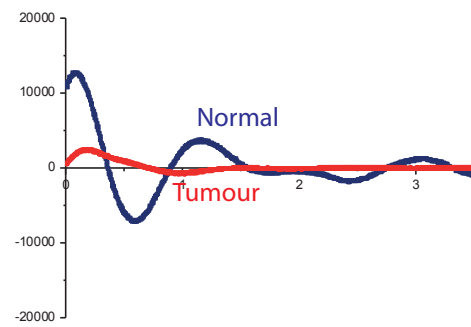

Supplement: Supplementary file 4 — Figure S4. Circadian clocks in breast tissue. Left - representative Per2::Luc traces from cultures of MECs isolated from the normal and tumour regions of patients with breast cancer. Right - normalisation of Per2::Luc activity from normal and tumour MECs. (PDF 287 kb) [file 13058_2018_1053_MOESM4_ESM.pdf]

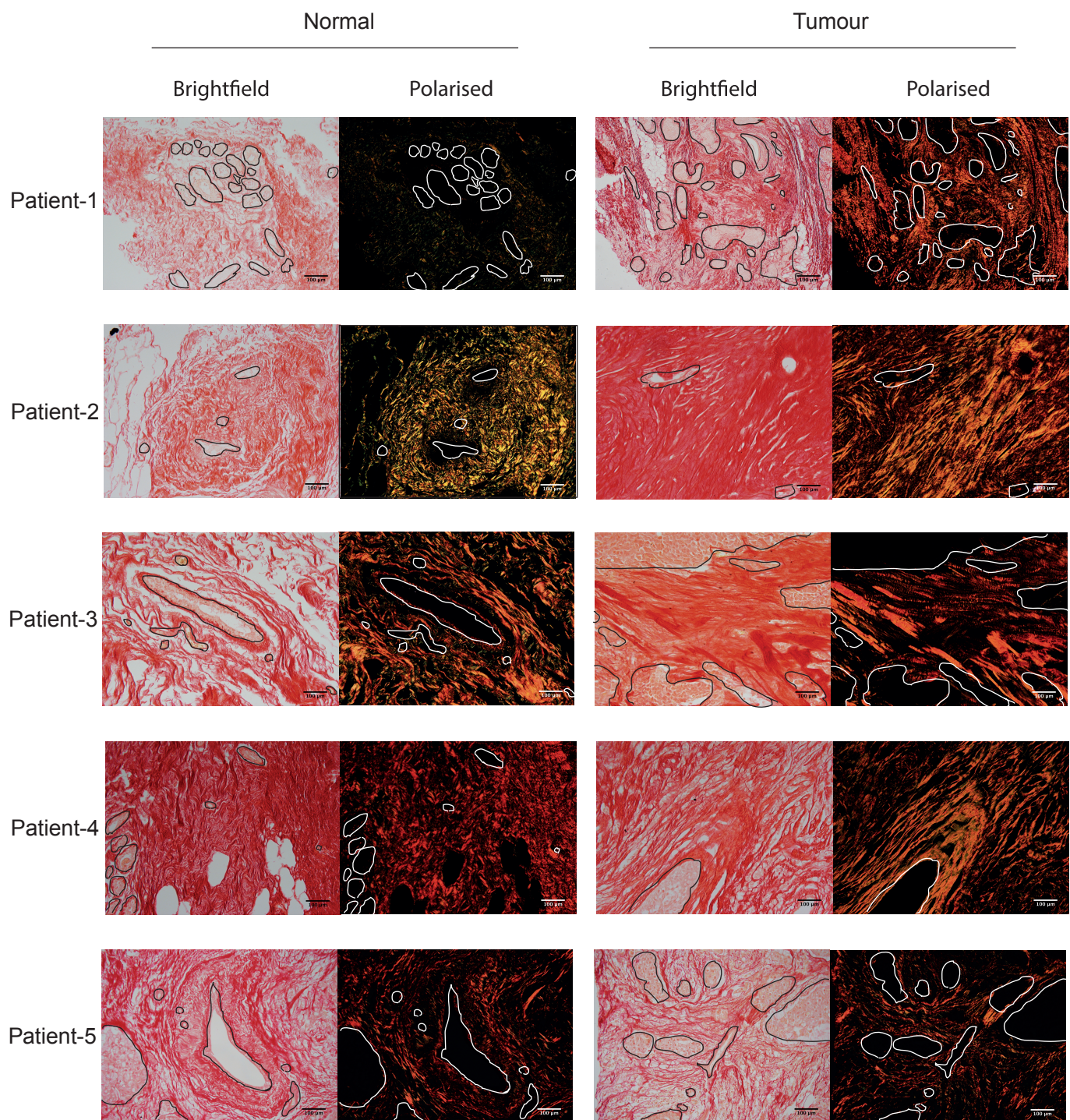

Supplement: Supplementary file 5 — Figure S5. Collagen organisation in normal and tumour stroma. Picrosirius-Red-stained paraffin sections visualised in bright-field or polarised light. Samples of normal (left) and tumour (right) tissue from the same individuals are shown in each case. Ducts are outlined in black and white. (PDF 82243 kb) [file 13058_2018_1053_MOESM5_ESM.pdf]

Normal

Tumour

Patient-3

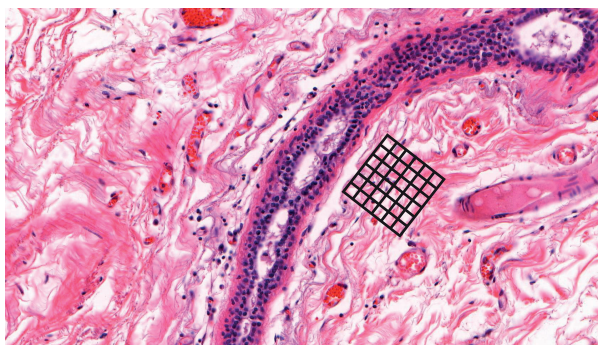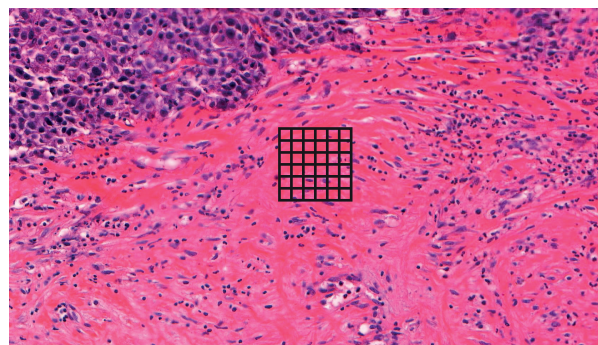

Patient-4

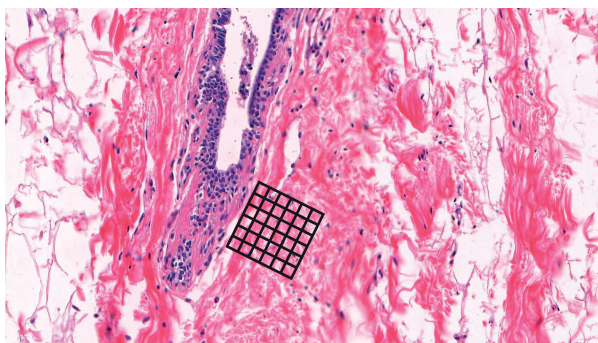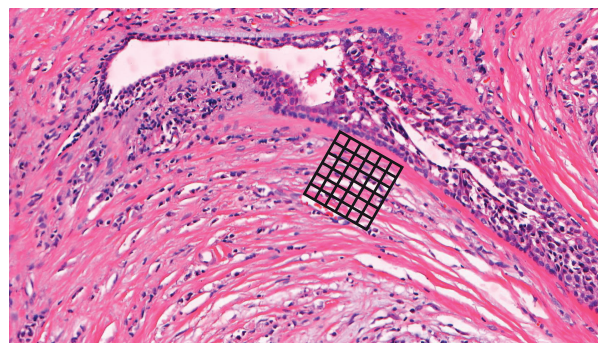

Patient-6

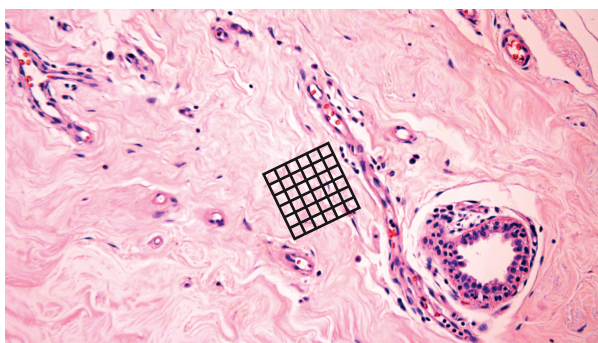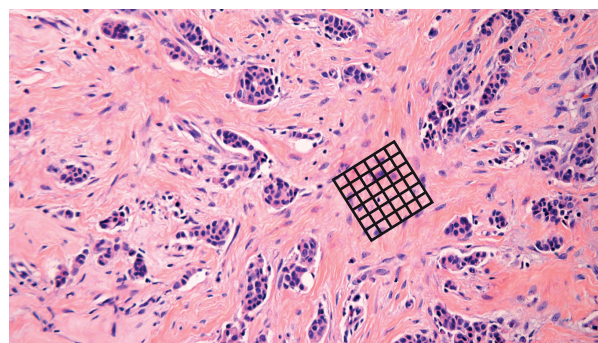

Patient-7

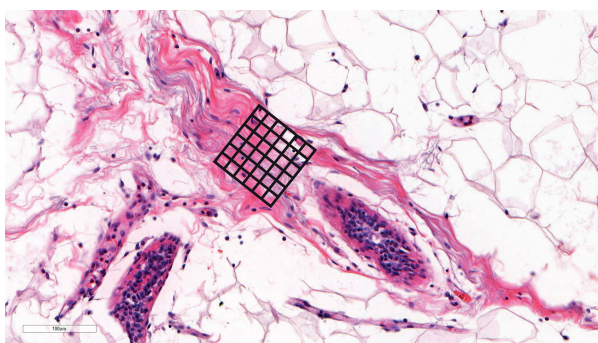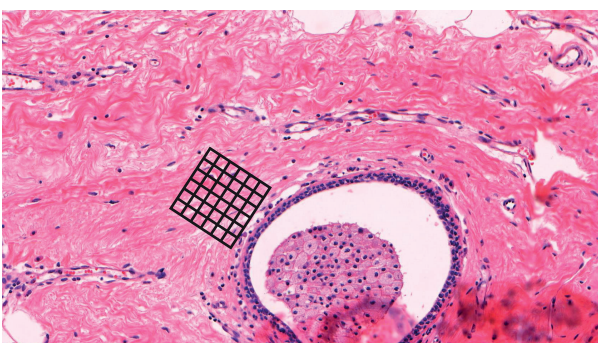

Patient-8

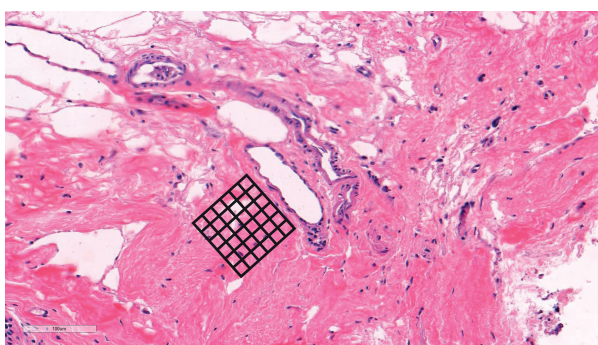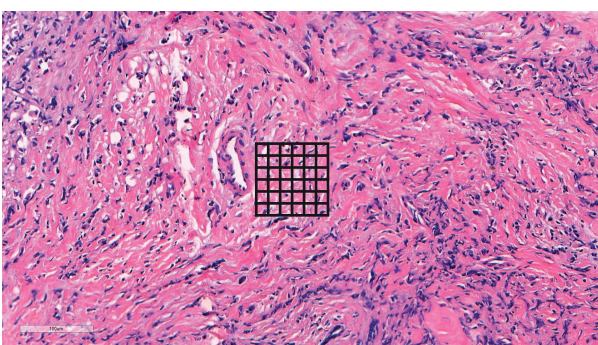

Supplement: Supplementary file 6 — Figure S6. Stromal regions analysed by AFM. H&E staining of the normal and tumour regions of breasts from each individual that were examined by AFM (see Fig. 4a). The black squares represent the exact regions that were analysed. (PDF 36538 kb) [file 13058_2018_1053_MOESM6_ESM.pdf]
